# Molecular mechanisms governing antifungal drug resistance

**DOI:** 10.1038/s44259-023-00007-2

**Published:** 2023-07-17

**Authors:** Yunjin Lee, Nicole Robbins, Leah E. Cowen

**Affiliations:** Department of Molecular Genetics, University of Toronto, Toronto, ON M5G 1M1 Canada

**Keywords:** Fungal evolution, Pathogens, Antimicrobial resistance

## Abstract

Fungal pathogens are a severe public health problem. The leading causative agents of systemic fungal infections include species from the *Candida*, *Cryptococcus*, and *Aspergillus* genera. As opportunistic pathogens, these fungi are generally harmless in healthy hosts; however, they can cause significant morbidity and mortality in immunocompromised patients. Despite the profound impact of pathogenic fungi on global human health, the current antifungal armamentarium is limited to only three major classes of drugs, all of which face complications, including host toxicity, unfavourable pharmacokinetics, or limited spectrum of activity. Further exacerbating this issue is the growing prevalence of antifungal-resistant infections and the emergence of multidrug-resistant pathogens. In this review, we discuss the diverse strategies employed by leading fungal pathogens to evolve antifungal resistance, including drug target alterations, enhanced drug efflux, and induction of cellular stress response pathways. Such mechanisms of resistance occur through diverse genetic alterations, including point mutations, aneuploidy formation, and epigenetic changes given the significant plasticity observed in many fungal genomes. Additionally, we highlight recent literature surrounding the mechanisms governing resistance in emerging multidrug-resistant pathogens including *Candida auris* and *Candida glabrata*. Advancing our knowledge of the molecular mechanisms by which fungi adapt to the challenge of antifungal exposure is imperative for designing therapeutic strategies to tackle the emerging threat of antifungal resistance.

## Introduction

Fungal pathogens are a significant threat to human health, infecting billions and taking the lives of approximately 1.5 million people annually^1^. Patient populations most susceptible to systemic fungal infections include immunocompromised persons; including those with HIV/AIDS, and patients receiving immunosuppressive agents, or undergoing cancer therapy^2^. The predominant aetiological agents of systemic fungal infections are *Candida*, *Cryptococcus*, and *Aspergillus* species^1^. Among *Candida* species, *Candida albicans* is the most common cause of invasive candidiasis globally. As a typical resident of the human microbiota, *C. albicans* can exist as a harmless commensal in healthy individuals; however, this fungus can cause life-threatening infections in immunocompromised patients with mortality rates exceeding 40% despite treatment^2^. *Cryptococcus* is the second leading cause of mortality in adults living with HIV in sub-Saharan Africa, with an estimated global fatality of 112,000 people every year^3^. Most cryptococcal infections are attributed to *Cryptococcus neoformans*, which can be acquired by inhaling infective airborne yeast cells^2^. *Aspergillus* species are ubiquitous moulds causing invasive aspergillosis, which carries a mortality rate of 50% even with proper diagnosis and intervention, and if left untreated, fatality can approach 100%^1^. Like *Cryptococcus*, the primary route of *Aspergillus* transmission is through inhalation of airborne spores, causing severe illnesses in people with weakened immune systems^2^. The primary causative agent of invasive aspergillosis is *Aspergillus fumigatus*, an organism naturally found in the soil as a saprotroph on organic debris^2^. Thus, the alarming impact of opportunistic fungi on global public health solicits a pressing need for effective therapeutic strategies to combat these life-threatening infections.

**Fig. 1:**
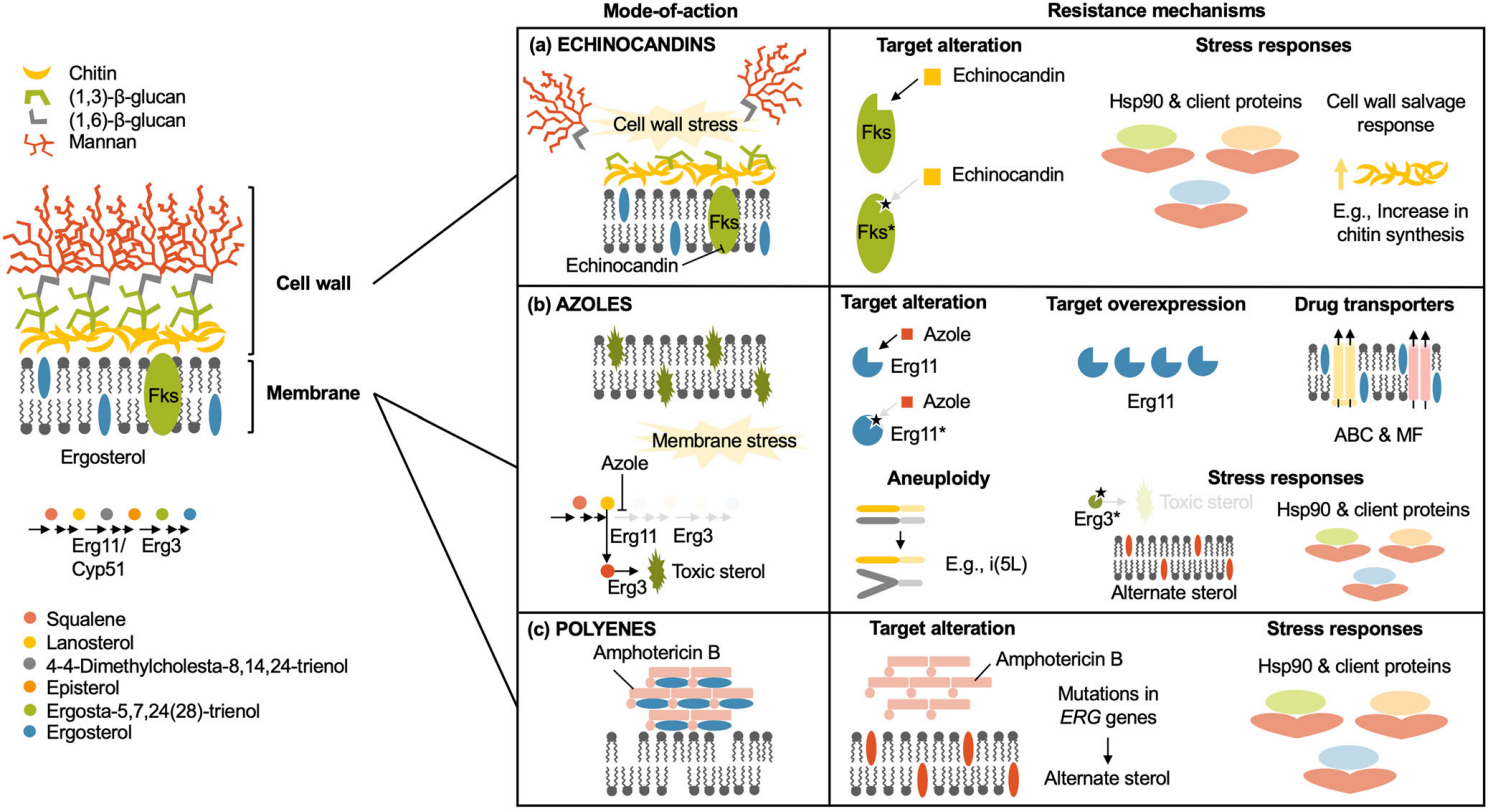
Antifungal mode-of-action and mechanisms of resistance. **a** The echinocandins function by inhibiting (1,3)-β-D-glucan synthase, disrupting cell wall integrity and causing severe cell wall stress. Echinocandin resistance is primarily mediated by mutations in the drug target gene, *FKS1*, in *Candida*, *Cryptococcus*, and *Aspergillus*. For *C. glabrata*, mutations occur in both *FKS1* and its paralogue *FKS2*. Cellular factors enabling responses to echinocandin-induced stress include the molecular chaperone Hsp90, various Hsp90 client proteins, and genes that regulate cell wall salvage signalling (e.g., compensatory upregulation of chitin synthesis). **b** The azoles act on the fungal cell membrane via the inhibition of lanosterol 14-α-demethylase (Erg11), which blocks ergosterol biosynthesis and results in the accumulation of a toxic sterol intermediate (14-α-methyl-3,6-diol) produced by Erg3. Azole resistance can arise through mutations in the drug target (*ERG11*) or target overexpression. Loss-of-function mutations in *ERG3* can also confer azole resistance by blocking toxic sterol accumulation and this mechanism is contingent on Hsp90 and its client proteins. Efflux is also a major azole resistance determinant and involves the upregulation of ABC and MF transporters. Aneuploidies such as a duplication of the left arm of chromosome 5 (termed isochromosome (i5(L))) can increase dosage of the azole target Erg11 and efflux pumps. **c** The polyene drug amphotericin B forms extramembranous aggregates that extract ergosterol from fungal cell membranes, acting as a sterol “sponge”. While resistance remains extremely rare, it can be acquired through mutations in ergosterol biosynthesis genes resulting in the depletion of ergosterol and the accumulation of alternate sterols. As with resistance to other antifungals, resistance to amphotericin B is also contingent on Hsp90-dependent stress responses.

The generation of antifungals has been slow-paced in part due to the close evolutionary relationship between fungi and humans, limiting the number of fungal-specific targets that can be exploited for drug development. Indeed, only three major classes of antifungal drugs exist in clinical practice to treat invasive fungal diseases (Fig. 1). The polyenes (e.g., amphotericin B) represent the oldest antifungal drugs used in medical practice to treat systemic fungal infections. These amphipathic molecules exert fungicidal activity by extracting ergosterol from lipid bilayers, akin to a “sterol sponge”^4^. While polyenes exhibit broad-spectrum bioactivity against yeast and mould species, their significant host toxicity disfavours their clinical use^5^. However, amphotericin B remains the treatment of choice in resource-limited settings, particularly for the treatment of infections caused by *C. neoformans*. Specifically, the combination regimen of amphotericin B with the antimetabolite pyrimidine analogue flucytosine is a first-line treatment for cryptococcal meningitis in HIV-infected patients^6^. The azoles (e.g., fluconazole) are a class of five-membered heterocyclic compounds, which have been widely deployed in the clinic for decades to treat systemic fungal infections. Like polyenes, the azoles also target the fungal cell membrane; however, azoles are fungistatic against *Candida* and work by inhibiting the cytochrome P450 lanosterol 14-α-demethylase (Erg11). This results in a block in ergosterol biosynthesis and the build-up of a toxic sterol produced by the Δ-5,6-desaturase (Erg3)^7^. While azoles are well tolerated, a significant drawback is their potential to disrupt the metabolism of other drugs, as they also inhibit mammalian cytochrome P450^5^. Finally, the echinocandins (e.g., caspofungin) are cyclic hexapeptides with lipid side chains that target the fungal cell wall via inhibition of (1,3)-β-D-glucan synthase. While echinocandins exert fungicidal and fungistatic activity against *Candida* and *Aspergillus*, respectively, this drug class is ineffective against *C. neoformans*^5^.

Alarmingly, our limited repertoire of antifungal drugs is further threatened by the emergence of drug-resistant isolates in the clinic. Resistance is readily acquired through drug target alteration or overexpression, enhanced efflux pump activity, or through activation of cellular stress response pathways (Fig. 1). Further compounding this public health threat is the increased prevalence of inherently drug-resistant fungi, such as the emerging non-*albicans Candida* species, *Candida auris* and *Candida glabrata*^8^. Thus, in light of the growing public health threat of drug-resistant pathogenic fungi, a thorough understanding of antifungal resistance mechanisms is vital for the management of infections caused by these microbes. Here, we summarize our current knowledge of the molecular mechanisms underlying resistance in major and emerging fungal pathogens.

## Drug target alteration or overexpression

Drug target alteration is a common mechanism leading to acquired antifungal resistance. For azoles, resistance involves alterations in genes encoding the azole drug target 14-α-demethylase (*ERG11* in yeast and *cyp51* in moulds). In clinical isolates of *C. albicans*, over 140 distinct Erg11 amino acid substitutions have been reported in the literature, with most clustering into three hot spot regions^9,10^. Structural analysis of *C. albicans* Erg11 demonstrated that while some substitutions involve residues that are exposed in the active-site cavity (directly affecting azole binding), others occur on the proximal surface of the enzyme and may indirectly contribute to azole resistance (for example, by influencing interactions with cytochrome P450 reductase)^11^. Mutations in *ERG11* also contribute to reduced azole susceptibility in the emerging fungal pathogen *C. auris*^12–15^, with a recent study highlighting a previously unreported substitution in Erg11 (F444L) identified in azole-resistant clade IV clinical isolates that has not been previously described in other reports^15^. For *C. neoformans*, a limited number of *ERG11* mutations have been described in azole-resistant clinical isolates^16^. Unlike *Candida* or *Cryptococcus*, *A. fumigatus* expresses two genes encoding Cyp51 isoenzymes, namely *cyp51A* and *cyp51B*, that are 60% identical. Mutation of *cyp51A* is a well-characterized and frequently-documented determinant for triazole resistance in *A. fumigatus*, however, the role of *cyp51B* mutations in azole resistance is less clear^17,18^. Recently, it was demonstrated that introduction of a G457S Cyp51B substitution, originally identified in a resistant clinical isolate, into a susceptible recipient conferred voriconazole resistance, reinforcing the notion that mutations in *cyp51B* should be monitored in the context of clinical resistance^17^. Alarmingly, duplications in the *cyp51A* gene promoter coupled with specific amino acid substitutions, TR34/L98H and TR46/Y121F/T289A, are becoming widespread in the environment associated with azole use in agriculture, while also isolated from hospital patients without previous exposure to antifungal drugs^19–22^. Recent genomic analysis of *A. fumigatus* isolates from the United States suggests a single introduction of a strain with the TR34/L98H substitution resulted in widespread distribution throughout susceptible *Aspergillus* populations through recombination between resistant and susceptible isolates^23^. Collectively, this growing body of literature suggests that environmental *Aspergillus* isolates serve as a reservoir for drug-resistant clinical infections around the globe.

Increased drug target expression is another prevalent mode of acquiring azole resistance. In azole-resistant clinical isolates of *C. albicans*, gain-of-function mutations in the zinc cluster transcription factor gene *UPC2* can lead to constitutive overexpression of *ERG11*^24^. Upc2 contains an N-terminal nuclear localization signal (NLS) and a C-terminal ligand-binding domain (LBD), the latter of which can sense cellular ergosterol levels^25^. The Upc2 LBD exhibits ligand-dependent conformational switching and mutations of the glycine-rich loop interfere with ligand binding via steric clashes, resulting in constitutive activation^26^. Intriguingly, recent work demonstrated an expansive role of *C. glabrata* Upc2A (*C. glabrata* homologue of *C. albicans* Upc2) in controlling the expression of genes beyond those involved in ergosterol biosynthesis, including those associated with translation and plasma membrane constituents^27^. While the roles of these target genes in mediating antifungal resistance remains enigmatic, it would be interesting to directly assess their impact(s) on azole susceptibility. In *A. fumigatus* and *C. neoformans*, expression of *cyp51A /ERG11* in response to azole exposure is regulated by the sterol regulatory element-binding protein (SREBP), SrbA and Sre1, respectively^28,29^. Additional regulators of *cyp51A* expression that contribute to azole resistance in *A. fumigatus* include the fungal-specific transcription factors AtrR and SltA^30–32^.

As with azoles, resistance to echinocandin therapy frequently involves mutations in genes encoding the drug target, *FKS1* (*Candida, Cryptococcus*, and *Aspergillus* spp.) and *FKS2* (*C. glabrata* only)^33,34^. In *C. albicans*, *FKS1* is an essential gene encoding a catalytic subunit of the 1,3-β-glucan synthase complex, whereas in *C. glabrata*, *FKS1* and *FKS2* are functionally redundant for viability^35^. A recent study leveraging experimental evolution with *C. glabrata* generated 121 anidulafungin-resistant lineages. Intriguingly, all 121 strains harboured non-synonymous mutations in *FKS* genes, with mutations preferentially occurring in *FKS2* as opposed to *FKS1*^36^. Although most mutations were identified in well-characterized hot-spot regions, 22% of *FKS* mutations were identified elsewhere in the open reading frame, highlighting the importance of monitoring the contribution of mutations across the entirety of the *FKS* genes as opposed to simply focusing on hot-spot regions. Several amino acid substitutions in *FKS1* have also been associated with decreased echinocandin susceptibility in *C. auris*^12,37–42^. Recently, through the use of cryo-electron microscopy with the model yeast *Saccharomyces cerevisiae*, structural analysis of echinocandin-resistant substitutions in Fks1 indicated three hot spot regions clustered at a region near transmembrane (TM) helices 5–6 and 8 which is surrounded by ordered lipid molecules^43^. Further analysis of the structure of mutant Fks1 (S643P), which affects lipid binding, revealed conformational changes that lead to shifts of nearby bound lipids, suggesting a potential additional mechanism for drug resistance that does not simply result in a decrease in drug binding to its target but that is a result of alterations in cellular physiology^43^. Additionally, the authors developed a radiolabel-free assay system for Fks1 activity, whereby activity is measured by the release of UDP. Together, this work lays the framework for future analyses of the structural implications of *FKS1* mutations in other fungal pathogens and provides a platform for the development of more effective inhibitors of this important antifungal target.

For *A. fumigatus*, echinocandin resistance in clinical settings is extremely rare, likely due to the fitness cost of *FKS1* mutations in vivo^44^. In a mouse model of invasive pulmonary aspergillosis, infection with an echinocandin-resistant strain of *A. fumigatus* was less lethal when compared with the echinocandin-susceptible strain, as demonstrated by reduced lung fungal burden and significantly longer survival time^44^. Notably, echinocandins retained some activity against the *A. fumigatus FKS1* mutant strain in vivo, albeit a lack of appreciable activity in vitro, which may further contribute to the rare clinical occurrence of echinocandin resistance in this species^44^.

Severe fitness trade-offs disfavour the acquisition of resistance to the polyene amphotericin B in multiple fungal species, explaining why resistance to this antifungal class remains extremely limited in the laboratory and clinic^45^. Resistance may arise from alterations in the ergosterol biosynthesis pathway, causing depletion of ergosterol and the accumulation of alternate sterols. In *Candida* species, amphotericin B resistance has been associated with mutations in several ergosterol biosynthesis genes, including *ERG2*, *ERG3*, *ERG6*, and *ERG11*^42,45–47^. However, such mutations that confer polyene resistance simultaneously create severe cellular stress, which results in hypersensitivity to oxidative stress, febrile temperatures, and killing by neutrophils^45^. In *C. neoformans*, despite the use of amphotericin B as the first line of defence against meningeal cryptococcosis, only one case of a resistant clinical isolate harbouring a mutation in *ERG2* has been documented to date^48^. Overall, target alteration is a canonical mechanism by which fungal pathogens evolve resistance, particularly to the azoles and echinocandins, representing a significant clinical challenge to antifungal therapy.

## Increased efflux

In addition to target alteration or overexpression, enhanced drug efflux is a dominant mechanism of resistance to diverse antimicrobials. Resistance to azole therapy often involves the upregulation of plasma membrane efflux pumps, which act to reduce intracellular drug accumulation. In contrast, efflux-mediated resistance mechanisms are not involved in adaptive resistance to echinocandins or polyenes, as they are poor pump substrates^49^. For *C. albicans*, upregulation of the ATP-binding cassette (ABC) transporters, Cdr1 and Cdr2 (*Candida* drug resistance 1 and 2), and major facilitator transporter Mdr1 (multidrug resistance 1) have been implicated in azole resistance^50,51^. Specifically, gain-of-function mutations in the transcriptional activator gene, *TAC1*, result in constitutive overexpression of both *CDR1* and *CDR2*, while activating mutations in *MRR1* are responsible for the upregulation of *MDR1*^52,53^. Likewise, gain-of-function mutations in *TAC1B* (*C. auris* homologue of *C. albicans TAC1*) and *MRR1A* (*C. auris* homologue of *C. albicans MRR1*) contribute to clinical fluconazole resistance in *C. auris*^54,55^. For example, a study examining 304 globally-distributed *C. auris* clinical isolates representing each of four of the major genetic clades identified three predominant *TAC1B* mutations, encoding A640V, A657V, and F862_N866del substitutions, from clades Ic, Ib, and IV, respectively^54^. Additional work demonstrated another *TAC1B* mutation (encoding S611P) capable of conferring enhanced fluconazole resistance among clade-IV clinical isolates, further highlighting that *TAC1B* mutations contribute to azole resistance across diverse clades^15^. In contrast, mutations in *MRR1A* appear to play a more restrictive role with resistance through an N647T substitution, which is reported specifically in clade III isolates^56^. In *C. glabrata*, the expression of *CDR1* is controlled by the zinc cluster transcription factor, Pdr1 (pleiotropic drug resistance 1)^57,58^. Recently, it was demonstrated that the zinc cluster transcription factor Upc2A also regulates *CDR1* expression and that extensive overlap exists between the regulatory networks mediated by Upc2A and Pdr1 in *C. glabrata*^27,59^. Overexpression of multidrug efflux transporters also mediates azole resistance in other fungal pathogens, with the major players being the ABC transporters Afr1 and AtrF for *C. neoformans* and *A. fumigatus*, respectively^60,61^. Additionally, in *A. fumigatus*, the fungal-specific Zn_2_-Cys_6_ type transcription factor AtrR plays a pivotal role in regulating the expression of *cyp51A*^30,31^. Thus, efflux-mediated resistance to azoles is active in diverse fungal pathogens, highlighting the potential for targeting drug efflux to overcome resistance.

## Genomic plasticity

In addition to specific point mutations leading to enhanced drug target or efflux pump expression, another mechanism by which this can occur in fungal pathogens is through genomic alterations. In the diploid organism *C. albicans*, the duplication of the left arm of chromosome 5 to form an isochromosome (i5(L)) is observed in azole-resistant clinical isolates, as well as laboratory-derived resistant strains^62^. This aneuploidy results in increased dosage of genes encoding both the azole target Erg11 and the transcription factor regulating drug efflux, Tac1^63^. Likewise, experimental evolution studies in *C. auris* suggest that an extra copy of chromosome 5 (which harbours several drug resistance-related genes) contributes to the development of fluconazole resistance in this pathogen^42,64^. Additionally, loss of heterozygosity (LOH) events can occur in specific genomic regions harbouring azole resistance determinants (*ERG11* and *TAC1*), rendering mutations acquired in these genes homozygous and thus increasing resistance^65^. Strikingly, changes in whole chromosome copy number can occur in response to diverse stress conditions and enable tolerance to antifungals despite no prior exposure. Recent work in *C. albicans* demonstrated that exposure to the cancer chemotherapeutic hydroxyurea, or the ER stress-inducing agent tunicamycin selects for trisomy of chromosome 2, which also enables tolerance to the echinocandin caspofungin^66,67^. Given that aneuploidy simultaneously changes the copy number of many genes at a given time, it has the potential to confer cross-tolerance to unrelated drugs.

Another significant source of genomic diversity that drives the rapid acquisition of azole resistance in *C. albicans* involves extensive copy number variations (CNVs), which amplify large genomic regions^68^. These complex CNVs are flanked by distinct long inverted repeat sequences and are found across the genome, often containing genes associated with drug resistance^68^. Interestingly, recent work observed that different antifungal drug concentrations can select for distinct genotypic and phenotypic outcomes in vitro. Through the evolution of 144 *C. albicans* lineages in the presence of various concentrations of fluconazole, the authors observed that those lineages evolved with drug concentrations close to their MIC_50_ (the minimum inhibitory concentration of drug that reduces growth by 50%) had a propensity to rapidly evolve an increased MIC_50_ and acquired distinct segmental aneuploidies and CNVs^69^. In contrast, lineages evolved with drug concentrations above their MIC_50_ acquired diverse mutational changes and increase in drug tolerance (the ability of a subpopulation of cells to grow above their MIC_50_)^69^. Although the mechanisms driving these distinct phenotypes remain to be investigated in detail, it was proposed that cells experience a different degree of stress depending on the drug concentration. In the presence of higher drug concentrations (supra-MIC), cells may slow down cell cycle progression to maintain viability, while cells may persist through cell cycle progression when exposed to lower drug concentrations, increasing the frequency of segmental aneuploidies. Furthermore, while mechanistic investigations into aneuploidy-mediated antifungal resistance have been undertaken with strains harbouring i(5L), further work is needed to characterize the genes contributing to resistance in isolates with other aneuploidies. In addition, future studies should explore different drug concentrations when evaluating the efficacy of antifungals, as well as the initial MIC of a clinical isolate, to improve treatment outcomes.

For the haploid yeast *C. glabrata*, genome rearrangements underlying azole resistance include chromosomal translocations, segmental duplications, and occasionally, the formation of *de novo* minichromosomes^70^. For *C. neoformans*, a phenomenon referred to as heteroresistance can lead to resistant populations. Here, single cells give rise to progeny with heterogeneous resistant phenotypes, with a small but consistent subset of progeny highly resistant to the azoles. This phenomenon allows populations to adapt to increasing concentrations of the azoles in a stepwise manner, although susceptibility is typically restored after passage in the absence of drug. In *C. neoformans*, heteroresistance is often acquired through the formation of disomies, the most prevalent being that of chromosome 1, which contains both *ERG11* and the ABC transporter gene *AFR1*^71^. Beyond genome rearrangements, horizontal gene transfer (HGT)-mediated antifungal resistance was recently demonstrated in *A. fumigatus*^72^. Notably, *A. fumigatus* can undergo both chromosomal and plasmid-mediated HGT of voriconazole resistance (via a mutated resistance-conferring allele of *cyp51A*), with the latter being detected under conditions of voriconazole stress alone, implicating HGT as a potential route to antifungal resistance in this pathogen^72^. Most fungal HGT events have been studied using *in silico* analyses, which ideally require the use of multiple independent methods to achieve a total evidence approach^73^. The experimental system and insights provided by this work lay the groundwork for future studies investigating the mechanism(s) underlying HGT in fungi. Beyond the stressors used in this study, it will be of interest to test HGT induction in additional host-relevant conditions to explore the extent to which HGT contributes to fungal pathogenicity. Thus, fungal pathogens possess remarkable genomic plasticity that promotes survival in different environments, with significant implications for the evolution of antifungal drug resistance.

## Cellular stress responses

**Fig. 2:**
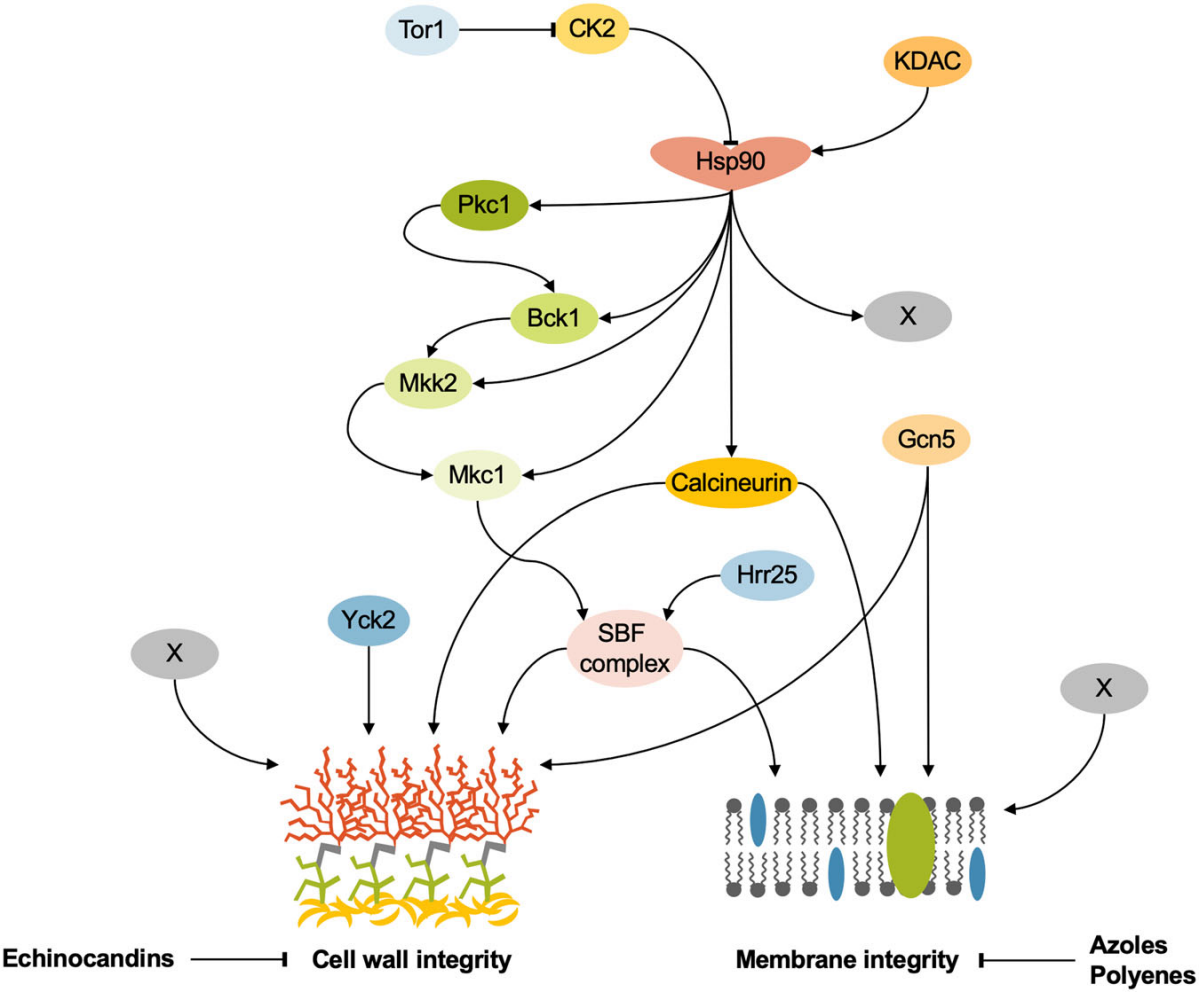
Cellular signalling governing responses to antifungal-induced stress. A global regulator of stress response circuitry is the molecular chaperone Hsp90. Hsp90 stabilizes key cell wall and membrane integrity regulators including the protein phosphatase calcineurin, PKC-MAPK pathway members, as well as other clients that remain to be identified. Hsp90 is subject to negative regulation by casein kinase 2 (CK2) and positive regulation by lysine deacetylases (KDACs). In addition to KDACs, lysine acetyltransferases such as Gcn5 have been implicated in antifungal-induced stress. Additional modulators of cell wall integrity include the casein kinase 1 (CK1) family members, Yck2 and Hrr25, with the latter kinase also serving as a regulator of cell membrane homoeostasis. Downstream of both Hrr25 and the PKC-MAPK cascade is the SBF transcription factor (Swi4/Swi6), which is an important mediator of both cell wall and membrane integrity. There are also several additional elusive factors that remain to be identified.

Cellular stress response pathways are critical in enabling pathogen survival in response to diverse environmental perturbations and, as such, play integral roles in alleviating antifungal-induced stress (Fig. 2). One central cellular hub mediating antifungal drug tolerance and resistance is the molecular chaperone, Hsp90, which stabilizes various signal transducers involved in enabling antifungal-induced stress in *Candida, Aspergillus*, and *Cryptococcus*^74,75^. In *C. albicans*, key client proteins of Hsp90 include the Ca^2+^-calmodulin-activated protein phosphatase calcineurin, and multiple members of the PKC-MAPK cell wall integrity cascade (Pkc1, Bck1, Mkk2 and Mkc1)^75–78^. Consequently, Hsp90 inhibition blocks the activation of calcineurin-dependent stress responses and PKC signalling, abrogating tolerance and resistance to the azole and echinocandin drugs^76,78^. Moreover, the emergence of polyene resistance in *Candida* is also contingent on Hsp90, highlighting the conserved role of this protein in the acquisition of resistance to diverse antifungals^45^. Recently, the importance of Hsp90 in mediating cellular responses to azoles was also described in *C. neoformans*. Through the generation of a strain where levels of *HSP90* could be repressed with a copper-repressible promoter, the authors showed depletion of *HSP90* increases susceptibility to azoles as well as other cellular stressors in this pathogen^79^. While this work provides a tractable genetic system to dissect Hsp90 function in *C. neoformans*, it will be useful to develop alternative conditional-repression systems to improve gene regulation and bypass toxicity associated with copper treatment alone^79^. The vast majority of essential genes in *C. neoformans* have yet to be interrogated due to the technical constraints of genetic manipulation in this organism. Advancements in genetic tools to study essential gene function will propel the search for antifungal targets to control cryptococcal infection.

Another critical mechanism of azole resistance that relies on stress responses involves mutations in the Δ-5,6-desaturase gene, *ERG3*. Specifically, loss-of-function mutations in *ERG3* lead to a block in the accumulation of the toxic sterol 14-α-methyl-3,6-diol, which would otherwise accumulate in response to azole-mediated Erg11 inhibition^7^. In *C. albicans*, *erg3*-dependent azole resistance is contingent on Hsp90, calcineurin, and Pkc1^75,78^. While the role of *ERG3* mutations in azole resistance has been less evident in *C. glabrata*, recent work demonstrated that mutations in *ERG3* can be acquired upon echinocandin exposure *in vitro* and confer cross-resistance to fluconazole^36^. Although questions remain as to why and how mutations in *ERG3* emerged upon treatment with the cell-wall targeting echinocandins, this work contributes to a growing body of literature describing this phenomenon. In the related pathogen *Candida parapsilosis*, mutations in *ERG3* have been reported in strains resistant to azoles and echinocandins^80^. Further, *ERG3* mutations have also been identified in *C. auris* following echinocandin exposure^42^. More detailed analysis of the mechanism(s) underpinning *erg3*-mediated cross-resistance has the potential to illuminate the evolution of multidrug resistance in these emerging pathogens, which is important given that echinocandin treatment is now recommended as a first-line of defence for treating invasive candidiasis, and thus these reported multi-drug resistant phenotypes have important clinical implications.

In the context of echinocandins, cell wall salvage responses play integral roles in withstanding stress induced by the inhibition of glucan synthase. In *C. albicans* and *A. fumigatus*, one central mechanism involves the compensatory upregulation of chitin, an essential fungal cell wall polysaccharide^81^. In response to caspofungin, elevated levels of cell wall chitin can lead to attenuated antifungal activity at high concentrations, partially resorting growth through a phenomenon termed the paradoxical growth effect (or Eagle effect)^81^. In *C. albicans*, this compensatory chitin response to caspofungin depends on the core stress response regulators PKC, calcineurin, and the high-osmolarity glycerol (HOG) MAP kinase^82^. For *A. fumigatus*, the chitin biosynthetic response is controlled, at least in part, by calcineurin-mediated transcriptional upregulation of chitin synthases^83^.

Pkc1 has been extensively studied in the model yeast *S. cerevisiae* for its essential role in cell wall integrity signalling^84^. In brief, cell wall stress signals are triggered through cell surface sensors to the Rho1 GTPase, which in turn activates Pkc1 and a linear cascade of MAP kinases. Effectors downstream of the PKC-MAPK cascade include the terminal transcription factors Rlm1 and SBF (Swi4/Swi6)^84^. In *S. cerevisiae*, Rlm1 is the major regulator of cell wall integrity and its transcriptional output includes genes involved in cell wall biogenesis, while SBF is a cell cycle transcription factor that regulates the transcription of cell wall-related genes including *FKS2* under conditions of cell wall stress^84^. As discussed previously, PKC also regulates membrane stress responses in *C. albicans* and this process is largely dependent on Rlm1^78^. In addition to Pkc1, several other kinases have been recognized for mediating antifungal drug resistance. One such kinase is the target of rapamycin (TOR), which plays a central role in nutrient sensing and metabolism in eukaryotes. In *C. albicans*, hyperactivation of TOR can be used to bypass azole toxicity^85^. TOR mediates this effect by activating Hsp90 via modulation of casein kinase 2 (CK2) activity, leading to calcineurin stabilization^85^. In line with this, rapamycin blocks azole resistance acquired by loss-of-function of Erg3, a mode of resistance dependent upon Hsp90^86^. The natural product beauvericin, which potentiates azole activity via both inhibition of TOR and multidrug efflux, is effective against diverse fungal pathogens including *C. albicans*, *C. neoformans*, and *A. fumigatus*^87^. More recently, the casein kinase 1 (CK1) family members, Yck2 and Hrr25, were implicated in *FKS1*-mediated echinocandin resistance in *C. albicans*^88,89^. Hrr25 was also demonstrated to be required for azole tolerance in a clinical isolate^89^. Further mechanistic investigations determined that Hrr25 maintains cell wall homoeostasis by interacting with the SBF (Swi4/Swi6) transcription factor^89^. Given that SBF is a well-characterized downstream effector of the PKC-MAPK cascade, this highlights the potential crosstalk between Hrr25 and Pkc1 signalling in regulating drug-induced stress responses^78,89^, and thus would be an interesting avenue of future study.

## Epigenetic regulation

There has been a growing appreciation for epigenetic factors mediating acquired antifungal drug resistance. One well-characterized chromatin-based modification involves the addition/removal of acetyl groups to/from the lysine residues of histone tails or other cellular proteins. For instance, the expression of lysine deacetylases *HDA1* and *RPD3* are upregulated in fluconazole-resistant *C. albicans* isolates, specifically in the early stages of resistance acquisition^90^. Furthermore, deacetylation is required for Hsp90 function, such that pharmacological or genetic inhibition of lysine deacetylases impedes the physical interaction between Hsp90 and calcineurin, thereby blocking the induction of key stress responses required for maintenance of Hsp90-dependent azole resistance^91^. Acetylation-dependent regulation of Hsp90 is also crucial for azole and echinocandin resistance in *A. fumigatus*, with the lysine 27 residue playing a vital role in this process^92^. Lysine acetyltransferases, such as Gcn5, also mediate antifungal tolerance and resistance in *Candida* species^93–95^. Notably, in *C. glabrata*, inhibition of Gcn5 minimized the emergence of *PDR1* gain-of-function alleles in evolution experiments with fluconazole, suggesting the importance of histone modifications not only in the maintenance of azole resistance but also in the acquisition of azole resistance^95^.

Another significant post-translational modification in histone proteins involves the addition/removal of methyl groups by histone methyltransferases and demethylases. Despite the integral role of histone methylation on gene regulation, its impact on resistance to antifungals has only been very recently explored. In *C. glabrata*, disruption of *RPH1*, which encodes a putative histone demethylase, causes increased sensitivity to fluconazole and reduced basal expression of the canonical azole resistance-related genes *PDR1* and *CDR1*^96^. In addition, the histone H3K4 methyltransferase Set1 modulates responses to azoles as it is necessary for azole-induced expression of ergosterol biosynthesis genes, including *ERG11* and *ERG3*^97^.

Beyond histone modifications, chromatin remodelling complexes have also been implicated in antifungal resistance. In *C. albicans*, the SWI/SNF chromatin remodelling complex is a crucial coactivator of the fluconazole resistance-related transcription factor Mrr1^98^. Deletion of *SNF2*, which encodes the SWI/SNF catalytic subunit, significantly reduces the upregulation of *MDR1* expression and fluconazole resistance in *MRR1* gain-of-function strains^98^. In *C. glabrata*, the SWI/SNF complex, along with the histone chaperone Rtt106, mediate azole resistance by driving the expression of the multidrug transporter genes, including *CDR1*^99^. Notably, Rtt106 and some SWI/SNF subunits are fungal-specific and thus may serve as promising therapeutic targets to overcome antifungal resistance in this important pathogen^99^.

In addition to chromatin modifications, fungi also display RNA-based modes of antifungal resistance. In *Mucor circinelloides* (causative agent of mucormycosis), RNAi (RNA interference)-mediated epimutations can confer antifungal resistance via degradation of mRNAs of drug target genes^100,101^. Additional work showed the RNAi-dependent epimutation pathway is inhibited by the non-canonical RdRP-dependent Dicer-independent silencing pathway under non-stress conditions^102^. In a systemic mouse model of *Mucor* infection, epimutation-mediated drug resistance can be rapidly induced and reverted over the course of the infection, and thus represents a potential clinically-relevant factor affecting drug resistance^103^. Future work should determine whether epimutation-mediated drug resistance can be generalized to other fungal pathogens with functional RNAi machinery, including *C. neoformans* and *A. fumigatus*. It is noteworthy that the canonical RNAi pathway has been lost in *C. albicans* and other budding yeast species; however, these organisms possess noncanonical RNAi proteins^104^.

Given the ubiquitous nature of RNAi, there has been growing interest in RNA-based therapeutics for the treatment of fungal infections, particularly in the management of crop diseases^105^. While RNA-based strategies for combatting fungal infections in humans have received far less attention, it offers an alternative strategy to conventional drugs. While significant challenges with the delivery of antifungal RNAs include fungal cell permeability and susceptibility to degradation by RNases in the host environment, several promising approaches are underway in related systems, which may be applied to the treatment of fungal infections^106^. Advancing our understanding of RNAi systems in human fungal pathogens is poised to reveal suitable targets that may be exploited for RNA-based therapeutics to combat mycotic disease.

## Concluding remarks

Our scarce antifungal arsenal, combined with the rising prevalence of drug-resistant fungal pathogens, represents a significant clinical challenge for the present and future. Beyond the overwhelming impact of fungi on humans, fungal infections also cause massive declines in crop production and wildlife viability, posing a growing threat to worldwide food security and ecosystem health^107^. Despite the challenges inherent to antifungal development, considerable progress has been made to expand our antifungal arsenal in recent years. Antifungal agents with distinct modes of action in development include olorofim (which blocks *de novo* pyrimidine synthesis), fosmanogepix (which blocks glycosylphosphatidylinositol (GPI) production), and ibrexafungerp (which inhibits glucan synthesis via a mechanism distinct of the echinocandins)^108^. Additionally, several agents within existing antifungal drug classes with improved pharmacokinetics and safety are currently in development, such as the echinocandin rezafungin, which possesses a prolonged half-life, and the tetrazole oteseconazole, which exhibits higher specificity for fungal CYP51, minimizing drug-drug interactions^108^.

**Fig. 3:**
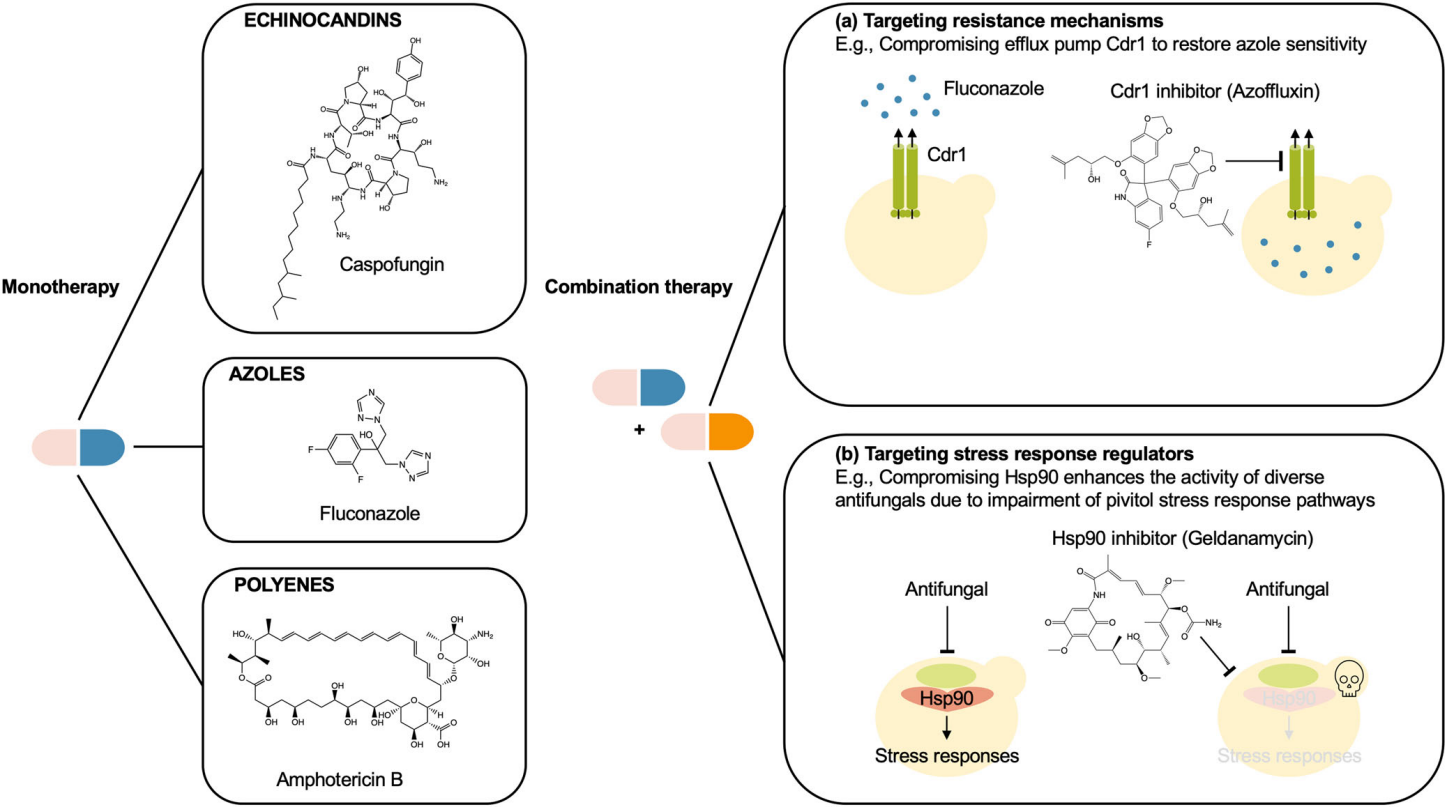
Combinatorial strategies to combat invasive fungal infections. Compared with monotherapy, treatment with drug combinations can improve drug efficacy and overcome resistance. **a** Targeting resistance mechanisms to improve antifungal efficacy due to increased bioavailability against multidrug-resistant pathogens. For example, pharmacological inhibition of efflux pump Cdr1 with a *bis*-benzodioxolylindolinone (azoffluxin) increases intracellular fluconazole levels, improving fluconazole activity against the emerging pathogen *C. auris*. **b** Targeting stress response regulators to enhance antifungal efficacy and impede the emergence of drug resistance. For example, pharmacological inhibition of Hsp90 with geldanamycin abrogates stress responses required to survive antifungal-induced stress.

The clinically-available armamentarium for antifungal treatment currently relies on targeting essential gene products or cellular processes in fungi. One promising alternative strategy to expand the antifungal target space and impede the evolution of resistance is the use of combination therapy (Fig. 3)^109^. Indeed, this approach has been proven efficacious for treating various infectious diseases such as malaria and AIDS/HIV^109^. Notably, recent work demonstrated that impairing efflux might be an effective strategy to restore azole sensitivity in the multidrug-resistant pathogens *C. auris* and *C. glabrata*^110,111^. In addition to resistance mechanisms, combination therapy can also involve the inhibition of critical regulatory hubs or modulators of cellular stress responses, such as the molecular chaperone Hsp90 and its client protein calcineurin (Fig. 3)^112,113^. While the conservation of such targets in humans poses challenges, structure-guided analyses can aid the design of fungal-specific inhibitors to minimize harmful effects on the host^114,115^. Beyond the optimization of existing drugs, high-throughput screening of compound libraries coupled with chemical genomic resources in fungal pathogens can facilitate the discovery of chemical matter to bolster the antifungal pipeline and enable the development of resistance-aversive therapies^116^. Continued efforts to advance our mechanistic understanding of antifungal resistance will undoubtedly support the development of therapeutic strategies to combat invasive fungal infections.

## Data Availability

The authors confirm that the data supporting the findings of this review are available within the article or are available from the corresponding author upon reasonable request.
